# Effectiveness of Various Treatments for Sudden Sensorineural Hearing Loss—A Retrospective Study

**DOI:** 10.3390/life12010096

**Published:** 2022-01-10

**Authors:** Magdalena B. Skarżyńska, Aleksandra Kołodziejak, Elżbieta Gos, Milaine Dominici Sanfis, Piotr H. Skarżyński

**Affiliations:** 1 Institute of Sensory Organs, Nadarzyn, 05830 Warsaw, Poland; p.skarzynski@csim.pl; 2 Center of Hearing and Speech Medincus, Nadarzyn, 05830 Warsaw, Poland; 3 World Hearing Center, Department of Teleaudiology and Screening, Institute of Physiology and Pathology of Hearing, Nadarzyn, 05830 Warsaw, Poland; a.kolodziejak@ifps.org.pl (A.K.); e.gos@ifps.org.pl (E.G.); 4 Child and Adolescent Health Program, Faculty of Medical Sciences, University of Campinas, Campinas 13083970, Brazil; msanfis@uol.com.br; 5 Heart Failure and Cardiac Rehabilitation Department, Faculty of Medicine, Medical University of Warsaw, 02091 Warsaw, Poland

**Keywords:** sudden sensorineural hearing loss, dexamethasone, prednisone

## Abstract

(1) Background: A retrospective clinical study was conducted to compare the effectiveness of different pharmacological and non-pharmacological regimens for treating sudden sensorineural hearing loss (SSNHL). (2) Methods: Adult patients (*n* = 130) diagnosed with sudden sensorineural hearing loss (SSNHL) and hospitalized between 2015 and 2020 were enrolled in this study. Depending on the treatment regimen applied, patients were divided into five groups. Inclusion criteria were as follows: (i) hearing loss of sudden onset; (ii) hearing loss of at least 30 dB at three consecutive frequencies; (iii) unilateral hearing loss; (iv) age above 18 years. Exclusion criteria were as follows: (i) no follow-up audiogram; (ii) bilateral hearing loss; (iii) recognized alternative diagnosis such as tumor, disorder of inner ear fluids, infection or inflammation, autoimmune disease, malformation, hematological disease, dialysis-dependent renal failure, postdural puncture syndrome, gene-related syndrome, mitochondrial disease; and (iv) age below 18 years. (3) Results: Complete recovery was found in 14% of patients (18/130) and marked improvement was found in 6% (8/130), giving an overall success rate of 20%. The best results were obtained in the second group (i.e., patients given intratympanic glucocorticoid + prolonged orally administered glucocorticoid) where the success rate was 28%. In general, the older the patient, the smaller the improvement in hearing, a correlation that was statistically significant. (4) Conclusions: In treating SSNHL, the highest rate of hearing recovery—28%—was in the group of patients given intratympanic corticoid plus prolonged treatment with orally administered glucocorticoid.

## 1. Introduction

Sudden sensorineural hearing loss (SSNHL) is, from a pathophysiological point of view, still poorly understood, even though first reports of it date back to 1944 [1]. SSNHL is defined as the sudden and rapid development (within 24 to 72 h) of hearing loss, in one or both ears, of at least 30 dB at three or more contiguous frequencies [2,3]. The hearing loss is often accompanied with tinnitus, dizziness, and a feeling of fullness in the ear.

The incidence of SSNHL is between 5 and 20 per 100,000. A German study has shown an incidence as high as 160 cases per 100,000 per year [4], although the true number is probably much higher. This is because many people do not seek medical help, and recovery occur by itself [5,6]. Many theories about the cause of the disease have been proposed. These include viral infections, immune disorders, blood flow disturbances, perforation of the eardrum, and metabolic or toxic causes [6,7].

Although numerous clinical and laboratory studies on SSNHL have been done, many diagnostic and therapeutic problems remain unresolved [8]. Cure for SSNHL is widely variable. Treatment regimens include inpatient and outpatient care. Applied separately or together, steroids, vitamins, hyperbaric oxygen, anticoagulants, histamine, diuretics, vasodilators, prostacyclin, hypervolemic hemodilution, tranquilizers, carbogen, and stellate ganglion block have all been reported to be effective in some cases [8,9]. It has been clinically demonstrated that treatment for SSNHL should start with an MRI of the head in order to exclude a tumor of the cerebellopontine angle or possible demyelination [5,7,10]. Laboratory tests are not routinely ordered, but more common tests such as complete blood counts, thyroid-stimulating hormone, clotting factors, erythrocyte sedimentation rate, and anticoagulants may be indicated [11]. It has been suggested that administration of intratympanic steroids may lead to higher drug concentrations in the inner ear than that achieved via the oral route and may be able to save hearing that is unresponsive to primary oral SSHNL steroid therapy [12]. The main aim of this study was to retrospectively investigate the effectiveness of different treatment regimens for SSNHL.

## 2. Materials and Methods

Between 2015 and 2020, 130 patients diagnosed with SSNHL were retrospectively selected from a database containing medical records from all patients admitted to the Institute of Physiology and Pathology of Hearing due to sudden sensorineural hearing loss (SSNHL) and enrolled to this study. The protocol of this retrospective study was approved by the Bioethics Committee of Institute of Physiology and Pathology of Hearing in Kajetany (IFPS:KB/Statement no. 17/2021). Inclusion and exclusion criteria were applied for patient selection, and patients were then assigned to one of five treatment groups. The inclusion criteria were as follows: (1) hearing loss of sudden onset; (2) hearing loss of at least 30 dB at three consecutive frequencies; (3) unilateral hearing loss; (4) age above 18 years. The exclusion criteria were as follows: (1) no follow-up audiogram; (2) bilateral hearing loss; (3) recognized alternative diagnosis such as tumor, disorder of inner ear fluids, infection or inflammation, autoimmune disease, malformation, hematological disease, dialysis-dependent renal failure, postdural puncture syndrome, gene-related syndrome, mitochondrial disease; and (4) age below 18 years. The allocation for the groups was based on the pharmacological or non-pharmacological treatment of SSNHL. Because we wanted to show the results of all patients, we did not excluded any patients who had full medical history and post SSNHL tests because different schemes of treatment were used for different patients, and the reasons for these may have depended on different factors. For example: contraindications for some pharmacological treatment, when the risk increases the benefit of the treatment. As a result, authors had no impact on the number of the participants in each group.

### 2.1. Patient Therapeutic Groups

The study was a retrospective analysis that included 130 patients who had been diagnosed with sudden sensorineural hearing loss between 2015 and 2020. Patients were divided into five groups according to the pharmacological or non-pharmacological treatment regimen that was used during the hospitalization. Therefore, the number of patients in each group varied. According to the retrospective medical data analysis, the first group of 30 patients (16 women and 14 men) were patients aged between 24 and 70 years (48.0 ± 14.9) treated with intratympanic glucocorticoid + orally administered glucocorticoid + hyperbaric oxygen therapy. The second group of 25 patients (14 women and 11 men) were patients who were treated with intratympanic glucocorticoid + prolonged orally administered glucocorticoid aged 18–84 years (51.7 ± 17.6). The third group consisted of 36 patients (15 women and 21 men) aged between 20 and 74 years (50.8 ± 14) who were treated with prolonged orally administered glucocorticoid and hyperbaric oxygen therapy. The fourth group comprised 30 patients (13 women and 17 men) aged 28–78 years (53.4 ± 14.8) who were treated only with prolonged orally administered glucocorticoid. The fifth group consisted of nine patients (five women and four men) who only received hyperbaric oxygen therapy as treatment (the only non-pharmacological treatment). 

**Figure 1:**
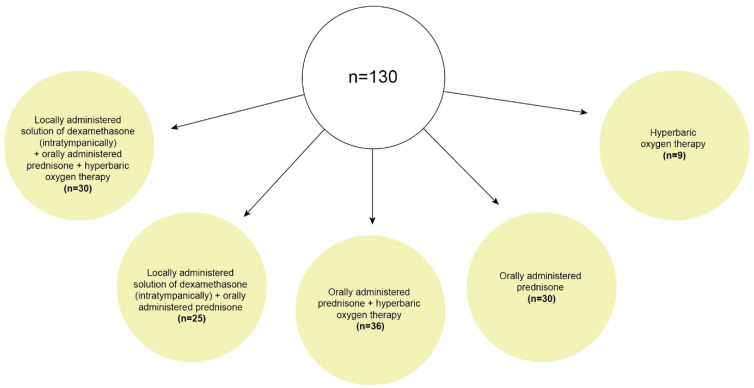
The patients were divided into five groups according to the type of treatment for SSNHL.

The five groups and comparisons between them are presented in Figure 1 and Table 1. 

Dexamethasone (brand name Dexaven^®^, solution for injection, concentration 4 mg/mL, 10 ampoules per package) and prednisone (brand name Encorton^®^, tablets, 1, 5, 10, and 20 mg per tablet) were administered to the patients. Table 2 summarizes the active substance, its concentration, pharmaceutical form, route of administration, and dose. The regimens, doses, and way of administering the drugs, which varied in accordance with the patient’s medical history, were as follows:

1. Dexamethasone sodium phosphate (4 mg/mL)—the maximum dose that was administered to patients was 2 mL (8 mg) once.
2. Prednisone—1 mg/kg body weight/24 h, orally in the morning.
3. Omeprazole —20 mg/24 h, orally in the morning during steroid therapy as a gastroprotective drug.

In Table 2, the information regarding the characteristics of medical products used in this study shows that that the prescribed dose was not exceeded.

### 2.2. Audiological Assessment

Audiological evaluation involved pure tone audiometry (PTA), with octave frequencies ranging from 125 to 8000 Hz. Measurements were made in the same soundproof cabin using the same diagnostic audiometer. PTA was performed on the day the patient came to the clinic and at a follow-up visit 3 months later on average (75% of diagnosed patients had a visit within 3 months). If there was no response at the maximum level at a given frequency, the hearing threshold was taken to be 5 dB greater than the maximum sound level generated by the audiometer. Additionally, the following tests were performed for diagnosis of SSNHL: (1) the patient’s medical history was established; (2) an otorhinolaryngological examination; (3) laboratory tests (CBC—complete blood count, prothrombin time, glucose level, lipid panel); (4) MRI with contrast in justified cases; (5) electrocardiogram (ECG); and (6) chest X-ray for patients qualified for hyperbaric oxygen therapy. 

### 2.3. Testing of Hearing

Hearing improvement was assessed using the criteria used by Labatut et al. (2013), who evaluated audiological outcomes after treatment by categorizing them into four groups in the same way as Furuhashi et al. (2002): (1) complete recovery, (2) marked improvement, (3) slight improvement, and (4) non-recovery [13,14]. Complete recovery or marked improvement were regarded as successful treatments. Table 3 shows the criteria for hearing improvement. 

Pure tone audiometry (PTA) was performed on the day the patient came to the hospital and again at the follow-up visit. The time between the initial diagnosis and the follow-up visit varied somewhat, but the average interval was 3 months (75% of diagnosed patients had follow-ups before 3 months). Table 4 shows the range, median, and standard deviation for each group. 

### 2.4. Statistical Analysis

Descriptive statistics and percentages were used to describe the characteristics of the study sample. Differences between hearing thresholds in the pre- and posttreatment period were assessed separately in each group through the Student test for paired samples. Analysis of variance (ANOVA) was used to compare posttreatment hearing thresholds across groups. Relationship between treatment delay, age, and treatment outcomes was assessed using *rho*-Spearman correlation coefficient. A *p*-value less than 0.05 was considered statistically significant. The analysis was conducted using IBM SPSS Statistics (v. 24).

## 3. Results

### 3.1. Characteristics of Patients

There were 130 patients enrolled in the study, 63 women and 67 men, aged from 18 to 84 years (*M* = 51.6; *SD* = 15.3). There were 72 patients who had SSNHL in the left ear and 58 in the right. Tinnitus was the most common symptom associated with SD, reported by 88 patients (68%). There were 40 patients (31%) who had dizziness and/or vertigo, and 34 (26%) reported feeling of fullness in the ear. The time interval from the onset of symptoms to the initiation of treatment (the treatment delay) ranged from 1 day to 16 days, with a mean delay of 5.1 days (SD = 3.5). Detailed demographic and clinical characteristics of the patients in the five groups are shown in Table 5. 

### 3.2. Hearing Improvement after Treatment 

Complete recovery was found in 14% of the patients (18/130) and marked improvement in 6% (8/130), giving a success rate of 20%. The best results were obtained in the second group (i.e., in the patients with intratympanic glucocorticoid + orally administered glucocorticoid), where the success rate was 28%. Detailed outcomes for all five groups are shown in Table 6. 

Pretreatment, the mean PTA in the first group was *M* = 65.7 dB (*SD* = 22.9), and after treatment, it improved to *M* = 56.4 dB (*SD* = 28.9); the mean change was 9.3 dB and was statistically significant (*t* = 2.66; *p* = 0.013). Significant improvement was also observed in the second group, from a pretreatment mean PTA of 58.1 dB (*SD* = 18.8) to a posttreatment mean of 42.4 dB (*SD* = 23.7); the mean change was 15.7 dB (*t* = 4.29; *p* < 0.001). The pretreatment mean PTA for the third group was *M* = 65.2 (*SD* = 24.9), and after treatment, it improved to *M* = 54.9 (*SD* = 26.4); the mean change was 10.3 dB and was statistically significant (*t* = 3.20; *p* = 0.003). Pretreatment, the mean PTA in the fourth group was *M* = 65.6 (*SD* = 28.4), and after treatment, it improved to *M* = 59.3 (*SD* = 29.8); the mean change was 6.2 dB and was statistically significant (*t* = 2.71; *p* = 0.011). An improvement was also observed in the fifth group, from the pretreatment mean PTA of *M* = 65.6 dB (*SD* = 28.4) to posttreatment *M* = 59.3 (*SD* = 29.8); however, here the mean change (10.0 dB) was statistically nonsignificant (*t* = 1.54; *p* = 0.161). 

Additionally, comparison was made between the final results of the five groups, using the ANOVA test. Its result was statistically non-significant: F = 1.53; *p* = 0.197.

**Figure 2:**
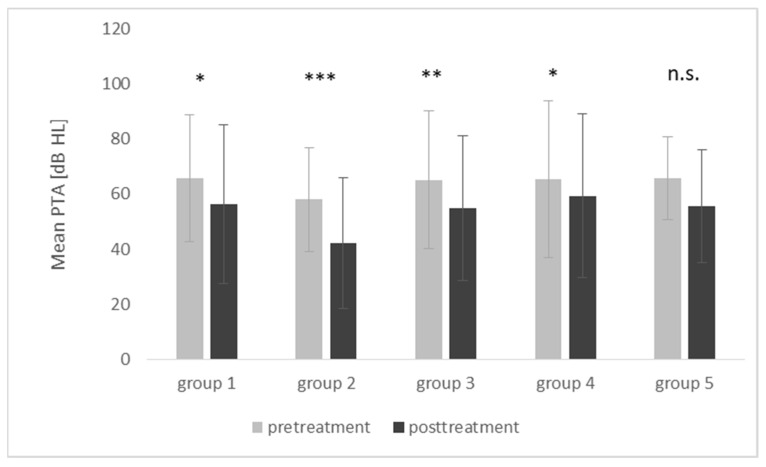
Pre- and posttreatment mean hearing thresholds for PTA in the five treatment groups. PTA—pure tone audiometry of 0.5, 1, 2, and 4 kHz; dB HL; the bars represent mean scores, the error bars represent standard deviations. *** *p* < 0.001; ** *p* < 0.01; * *p* < 0.05; n.s., not significant.

The pre- and posttreatment mean hearing thresholds are shown in Figure 2. The difference in the fifth group in comparison with the other groups may be due to the discrepancies and the small number of subjects in group 5.

### 3.3. Relationship between Treatment Delay, Age, and Treatment Outcomes 

**Figure 3:**
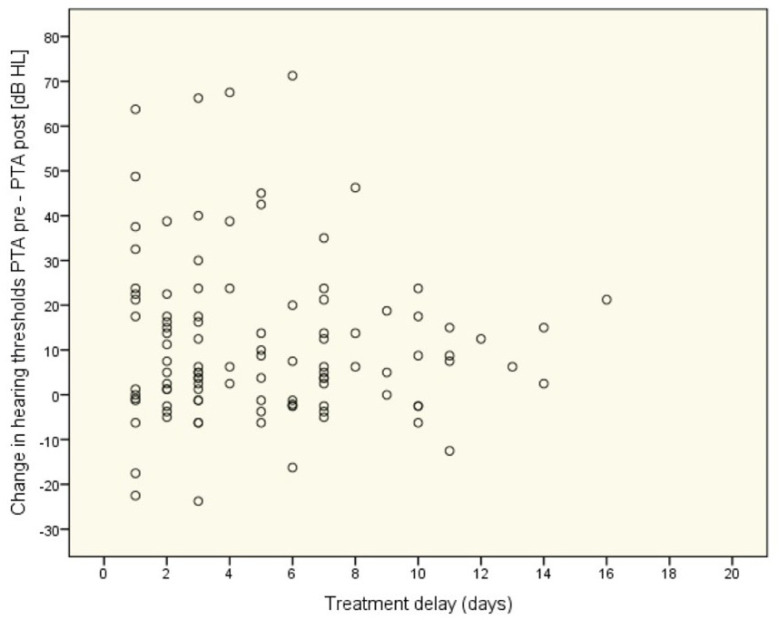
Relationship between treatment delay and change in hearing threshold (all patients, *n* = 130).

We assessed whether there was a relationship between the time interval from the onset of symptoms to the initiation of treatment (treatment delay) and the results of treatment. The change between PTA pre- and posttreatment was calculated for each participant (the bigger the change, the better the result of treatment) and was correlated with treatment delay (in days) in terms of a *rho*-Spearman correlation coefficient. For all patients, the correlation was statistically nonsignificant (*rho* = −0.03; *p* = 0.785), and in each of the five groups, it was also weak and statistically nonsignificant. The scatterplot in Figure 3 shows the relationship. 

**Figure 4:**
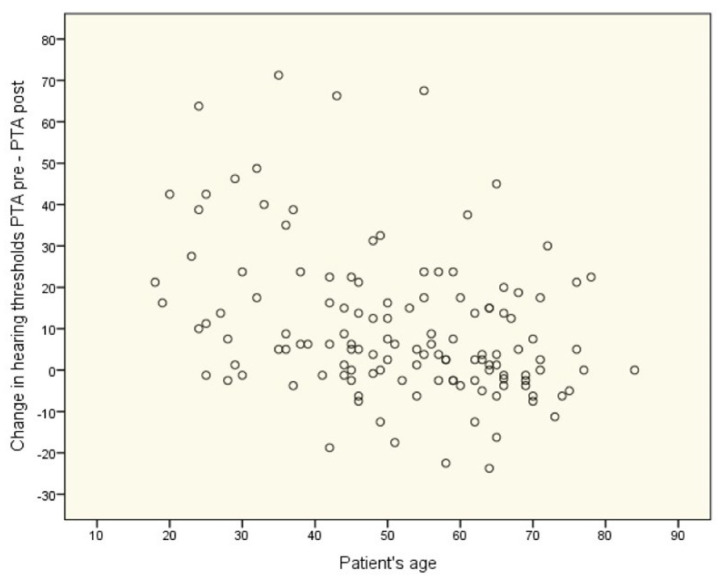
Relationship between age and change in hearing thresholds (all patients, *n* = 130).

On the other hand, the correlation between age and treatment outcome was statistically significant (*rho* = −0.34; *p* < 0.001). The older the patient, the less their hearing improved. Within the treatment groups, correlations were as follows: in the first group, *rho* = −0.46; *p* = 0.010; in the second group, *rho* = −0.21; *p* = 0.314; in the third group, *rho* = −0.12; *p* = 0.471; in the fourth group, *rho* = −0.43; *p* = 0.018; in the fifth group, *rho* = −0.69; *p* = 0.038. Figure 4 shows the data as a scatterplot. 

## 4. Discussion

Prognosis for recovery from sudden sensorineural hearing loss (SSNHL) depends on a number of factors such as: (1) the duration of symptoms; (2) specific impact on cochlear structures; (3) the disease process and associated symptoms; (4) the type and time of established pharmacological and non-pharmacological treatments; (5) demographic factors; and (6) audiogram characteristics [15,16]. In many cases, due to the underlying pathologic process, hearing will not improve, despite implementation of appropriate treatment [17,18,19]. On the other hand, according to the published clinical data, from 45% to 65% of patients who suffer from SSNHL will regain preloss hearing thresholds, even without therapy [16]. Numerous pharmacological agents have been investigated for the treatment of SSNHL—antimicrobials, vitamins, essential minerals, calcium antagonists, diuretics, defibrinogenators, vasodilators, volume expanders, anti-inflammatory agents—and there are still differing opinions because of the variety of treatment options, etiologies, rarity of the condition, and the lack of a gold standard for treatment [16]. According to a systematic review of 21 randomized controlled trials (RCTs) published between January 1966 and February 2006, no valid RCT has been done to determine the effectiveness of pharmacological or non-pharmacological treatments for SSNHL [9]. Only 2 of the 21 RCTs used identical criteria for the condition. Nonetheless, positive results were reported from administering systemic or intratympanic steroids, magnesium, vitamin E, batroxobin, and hyperbaric oxygen; at the same time, no difference was reported in audiometric outcomes after administering hemodilution agents and antivirals, and no difference was observed between placebo and systematic corticoids [9]. As a result, many different pharmacological and non-pharmacological options are available. For patients who suffer from hearing loss after an episode of SSNHL, cochlear implantation may be the only opportunity, due to the possible ossification of the cochlea—especially after viral infections [20,21,22,23]. As far as diagnostics of moderate to profound sensorineural hearing loss (MPSHL), the use of cVEMPs (cervical vestibular-evoked myogenic potentials) to identify and intervene promptly in cases of vestibular disorders, which could endanger the process of integration of the critical sensory stimuli for correct posture and locomotion [24,25]. Additionally, the evidence for viral involvement in SSNHL, Ménière’s disease, and vestibular neuritis is indirect and equivocal [26,27].

Glucocorticoids are classified according to Anatomical Therapeutic Chemical Classification System as H02AB02. Dexamethasone is a synthetic glucocorticoid with very strong and long-lasting pharmacological anti-inflammatory, immunomodulating, and anti-allergic activity. The strength of anti-inflammatory activity of dexamethasone is 7.5 times higher in comparison to that of prednisone and prednisolone and 30 times higher in comparison to hydrocortisonum. Two glucocorticoids with the highest anti-inflammatory activity are dexamethasone and betamethasone. Prednisone, prednisolone, triamcinolone, and 6a-methylprednisone have 4–5 times stronger anti-inflammatory activity than cortisol. Moreover, they all have a longer half-life than cortisol.

After intravenous administration, dexamethasone penetrates the cell membrane and binds with an inactive specific receptor in the matrix of the cell. This reaction activates the receptor, and the glucocorticoid–glucocorticoid receptor complex is translocated to a cell nucleus and binds with DNA. There, it gets into interaction with the specific short DNA sequences called glucocorticoid responsive elements (GREs), which enable gene transcription induction. It is a complex process due to the interaction with particular cofactors and proteins [28]. A negative response to a glucocorticoid is also possible. Webster et al. have reported identifying the genes that are negatively regulated by glucocorticoids. An example of a negative, so-called downregulation is the inhibition of the expression of genes responsible for encoding cytokines or enzymes (e.g., collagenase), which both play an essential role in immune and inflammatory reactions. Downregulation plays a crucial role in the immunosuppressive and anti-inflammatory effects of glucocorticoids [29]. After oral administration, the biological availability of prednisone is between 70% to 90%. The maximum concentration in plasma is achieved between 1 to 2 h, and the half-life is equal to 3.4 to 3.8 h in the plasma and 18–36 h in tissues. The protein binding (globulins and albumins) in the blood is on the level of 70–73%. From the pharmacological point of view, the pharmacokinetic and pharmacodynamic aspects of drugs are very important in the analyses of the properties of the drug and the boundaries and challenges in effective drug administration in the inner ear diseases that are being discussed. Prednisone, as an inactive medical substance, changes into an active metabolite prednisolone. The distribution volume of free drug fraction is equal to 0.86–1.81 L/kg mc. The main organ of metabolism of prednisone is the liver and to a lesser extent the kidneys. The elimination process is through bile and 1% to 5% with urine as inactive metabolites and in 10–20% as prednisolone. Dexamethasone after intravenous administration achieves the maximum concentration in the blood after 10–30 min and in the muscles after 60 min. The half-life of dexamethasone (t ½) is between 2.2 and 3.8 h. The metabolism of dexamethasone is in the liver and elimination is through bile. Additionally, 2.6% of unchanged chemical substance is eliminated by the kidneys. The main challenges in the administration of drugs systemically, not locally, is the presence of the blood–labyrinthine barrier in the inner ear, low local vascularization, and risk of side effects of systemic administration. The barrier and limitation of intracochlear administration is that there is no drug approved by the FDA (Food and Drug Administration) and the EMA (European Medical Agency) for intracochlear administration. Nevertheless, this method of drug administration is very important for the treatment of inner ear diseases due to no anatomical barriers. On the other hand, this way of administration is very invasive. The intratympanic administration has two barriers: (1) the presence of round and oval window and (2) clearance through the Eustachian tube [30].

Intratympanic or systemic corticoid therapy, off-label, with or without additional treatment, is currently the mainstay of treatment for idiopathic SSNHL in the United States. The prognosis for hearing recovery after idiopathic SSNHL depends on numerous factors such as age, presence of vertigo, severity of hearing loss, and the shape of audiogram [16]. Intratympanic corticoids are frequently used to treat SSNHL. This approach avoids potential adverse reactions of systemic administration, especially in patients with contraindications or who have failed to respond to systemic steroid administration; it also allows higher local concentrations of corticoid in perilymph to be achieved [17,31,32,33,34]. 

Similar results were obtained in this study. Complete hearing recovery was found in 14% of all patients (18 of the 130 enrolled in the study), and marked improvement was found in another 6% (8 of 130). That is, the overall success rate was 20% regardless of the treatment. The best rate of hearing recovery—complete recovery in 24%—came from the second subgroup of patients (*n* = 25), who received a solution of dexamethasone intratympanically (locally) followed by orally administered prednisone. Comparing this figure with the other four groups, the results progressively declined: group 1 = 13.3%, group 3 = 8.3%, group 4 = 16.7%. Looked at another way, in the second group, the rate of non-recovery was the smallest (40%) in comparison with all the other groups of patients; for comparison, the percentages of non-recovery cases were, in order, group 1 = 60%; group 3 = 63.9%; group 4 = 66.7%, and group 5 = 66.7%. Finally, in this study the correlation between treatment delay and change in PTA before and after treatment was not statistically significant, suggesting that treatment delay does not have a clinically important impact on the final outcome. According to the outcomes from all five groups of analyzed patients and their results, pharmacological treatment (steroids administrated locally or/and systemically) is more beneficial than non-pharmacological treatment only. Hyperbaric therapy alone was applied as one of the treatments in this study due to the contraindications for steroid administration in certain patients. Therefore, the number of patients in the last group was limited, only nine patients. 

According to the Cochrane library report from 2013 about the administration of the steroids in SSNHL, in the authors’ conclusion, the value of the steroids in the treatment of idiopathic sudden sensorineural hearing loss remains unclear since the evidence from the randomized controlled trials is contradictory in outcome, due to the small number of patients [35]. The clinical crucial effect of the using steroids in SSNHL is in salvage treatment in patients with SSNHL, but there is still room for additional studies, due to the poor quality of the component trials [36]. According to the pharmacotherapy in SSNHL in recommendations from 2019, antivirals, thrombolytics, vasodilators, and vasoactive substances should not be routinely prescribed. Corticosteroids as an initial therapy to patients may be offered within 2 weeks of the onset of symptoms and intratympanic steroids may be administrated when patients have incomplete recovery from sudden sensorineural hearing loss between 2 and 6 weeks after onset of symptoms of SSNHL [37]. Administration of glucocorticoids should be recommended for treatment of patients with SSNHL, especially in patients with SSNHL in the lower and middle frequency range, and those with pancochlear hearing loss have significantly better recovery of hearing levels [38]. According to the prospective randomized controlled clinical trial, where three different steroid therapies were assessed (oral steroid for 10 days in comparison with the intratympanic dexamethasone administration and in comparison with oral and intratympanic administration simultaneously), the conclusion was that all three treatment protocols resulted in the similar recovery rates [39]. In another study, hyperbaric oxygen therapy additional to systemic plus intratympanic steroid treatment did not affect hearing gain at all degrees of hearing loss, and in this study, the audiogram type and admission time did not affect hearing gain between the two groups [40]. The rate of non- recovery was the smallest in the second group (40%), and the rate of non-recovery in all other groups was similar, i.e., between 60% and 66.7%. According to the publication, the non-recovery rate may vary and depends on many factors such as: (1) early presentation (24 h), (2) associated tinnitus, (3) associated vertigo, and (4) comorbidities (diabetes, hypertension) [41]. In this study, the correlation between treatment delay and change in PTA before and after treatment was not statistically significant, which suggests that treatment delay does not have a clinically important impact on the final outcome. Till now, there is no one scheme of treatment that could be applied to all patients, due to the source and reason for onset of SSNHL. A maximum of 32–65% of cases of SSNHL may recover spontaneously, but according to the clinical specialists in the otorhinolaryngology field, the recovery rate may be overestimated [41]. 

### Limitations of the Study

A limitation of this study was the variable delays in treatment, which means there were different time intervals from the first clinically identified symptoms to the initiation of treatment. This delay ranged from 1 to 16 days, with a mean of 5.1 days. However, we found that the correlation between change in PTA (pretreatment compared to posttreatment) and treatment delay was statistically nonsignificant. Another limitation of this study was the discrepancy between the number of patients from the first four groups and the last one (the fifth group, *n* = 9).

## 5. Conclusions

According to the data from this retrospective analysis, complete recovery of hearing was found in 14% of patients (18/130) and marked improvement was found in 6% (8/130), giving a success rate of 20%. The best results in hearing recovery were observed in the second subgroup, where patients were given intratympanic corticoid + prolonged orally administered corticoid, giving a success rate of 28%. The correlation between age and the results of treatment was statistically significant and showed that the older the patient, the less likely was their hearing to improve. We found that the correlation between treatment delay and change in PTA from pretreatment and posttreatment was statistically nonsignificant.

## Figures and Tables

**Table 1:** Comparison between the five groups of patients according to the type of treatment for SSNHL.

	Group No 1	Group No 2	Group No 3	Group No 4	Group No 5
Dexamethasoni natrii phosphas (intratympanically)	X	X			
Prednisonum (orally)	X	X	X	X	
Hyperbaric oxygen therapy	X		X		X

**Table 2:** Summaries of product characteristics.

Active Substance	Concentraction	Pharmaceutical Form	Route of Administration	Dosage	Usage
Dexamethasoni natrii phosphas Prednisonum	4 mg/mL 1 mg/tablet 5 mg/tablet 10 mg/tablet 20 mg/tablet	Solution Tablet	Intravenously Orally	Between 4 and 16 mg/24h Exceptionally: 32 mg/24h 5–60 mg/24h Maximum: 250 mg/24h	Off-label use: Transtympanic Injection Via drain: 0.3 mL (1.2 mg) during meal

**Table 3:** Criteria for hearing improvement according to the criteria used by Labatut et al. [14].

	Value
Complete recovery	PTA < 25 dB HL
Marked improvement	PTA improvement > 30 dB HL
Slight improvement	PTA improvement 10–30 dB HL
Non-recovery	PTA improvement < 10 dB HL

PTA—puretone average of 0.5, 1, 2, and 4 kH; dB HL – decibels hearing level.

**Table 4:** The number of days between the onset of disease and follow-up visit in each group, showing range, mean (M), and standard deviation (SD).

Groups	Follow-Up (Days), Range: M (SD)
1.	8–570; 93.8 (141.9)
2.	8–561; 79.1 (129.1)
3.	4–548; 82.2 (109.2)
4.	10–371; 92.7 (99.1)
5.	7–113; 46.3 (34.4)

**Table 5:** Characteristics of the five patient groups. F—female, M—male, L—left, R—right, SD—standard deviation, M—mean.

Group	*n*	Age, Range: M (SD)	Sex F:M	Ear L:R	Feeling of Fullness in the Ear	Tinnitus	Dizziness/ Vertigo	Treatment Delay, Range; M (SD)
1.	30	24–70; 48.0 (14.9)	16:14	19:11	7 (23%)	16 (53%)	8 (27%)	1–13; M = 4.2 (3.4)
2.	25	18–84; 51.7 (17.6)	14:11	16:9	6 (24%)	19 (76%)	8 (32%)	1–16; M = 5.0 (3.8)
3.	36	20–74; 50.8 (14.0)	15:21	17:19	8 (22%)	22 (61%)	11 (31%)	1–14; M = 6.5 (3.9)
4.	30	28–78; 53.4 (14.8)	13:17	17:13	11 (37%)	24 (80%)	8 (27%)	1–10; M = 4.5 (2.6)
5.	9	29–77; 60.4 (15.5)	5:4	3:6	2 (22%)	7 (78%)	5 (56%)	1–8; M = 4.7 (3.0)

**Table 6:** Hearing improvement outcomes.

Group	Complete Recovery	Marked Improvement	Slight Improvement	Non-Recovery
1.	4 (13.3)	1 (3.3)	7 (23.4)	18 (60)
2.	6 (24.0)	1 (4.0)	8 (32.0)	10 (40.0)
3.	3 (8.3)	3 (8.3)	7 (19.5)	23 (63.9)
4.	5 (16.7)	1 (3.3)	4 (13.3)	20 (66.7)
5.	0 (0.0)	2 (22.2)	1 (11.1)	6 (66.7)

Data shown as the number of the patients; percentages are given in brackets.

## Data Availability

The data presented in this study are available on request from the corresponding author. The data are not publicly available due to protection of personal medical data.
